# Nutritional assessment in chronic kidney disease: the protagonism of longitudinal measurement

**DOI:** 10.1590/2175-8239-JBN-2020-0010

**Published:** 2020-03-23

**Authors:** Maria Ayako Kamimura, Fabiana Baggio Nerbass

**Affiliations:** 1 Universidade Federal de São Paulo, São Paulo, SP, Brasil; 2 Fundação Pró-Rim, Joinville, SC, Brasil

The importance of assessing nutritional status in patients with chronic kidney disease (CKD) is unquestionable due to its known close relationship with hard outcomes at all stages of the disease. Since there is no universal marker of nutritional status, measurement of anthropometric, biochemical, food consumption, and, more recently, functional capacity parameters are widely recommended.

In the presence of CKD, a constellation of factors can influence not only the nutritional status but also the susceptibility of the methods used. We can highlight hydration, inflammation, dialysis procedure, the limitations of dietary surveys, as well as observers’ variations, among others. Also, as the cut-off points for adequacy established for the general population are not always appropriate for these patients, the comparison of individuals over time takes a central role. Therefore, it is essential to establish a routine to monitor the nutritional status of CKD patients.

In this sense, the study by Oliveira et al.^1^ published in BJN aimed to monitor several nutritional parameters in a cohort of patients (14 on hemodialysis and 21 on peritoneal dialysis). The first assessment was carried out in the pre-dialysis phase (with a glomerular filtration rate <15 mL/min/1.73 m2); the second, at the beginning of dialysis treatment (2-4 days after the 1st dialysis session); and the third, after 30 days of treatment. The authors found that most variables remained constant during the follow-up with some significant differences mainly in the biochemical parameters. There was an increase in C-reactive protein levels, which was accompanied by a reduction in serum albumin levels and a decrease in mid arm muscle circumference. The increase in the protein equivalent of nitrogen appearance (PNA) certainly reflected a catabolic state in this period in a more important way than an increase in protein intake, as mentioned by the authors. These findings are in line with the literature concepts, and the concomitant changes were seen due to the longitudinal character of the study.

Considering that the metabolic pathways used during protein catabolism have high energy expenditure, it is reasonable to assume that the energy expenditure of these patients could have increased. However, in the 35 patients in the study, the resting energy expenditure (REE) measured by indirect calorimetry remained unchanged in the three evaluated moments. When Ikizler et al.^2^ investigated REE by direct calorimetry during and 2 hours after hemodialysis, the authors found that the REE was higher in the initial 2 hours of the session. In general, studies that evaluated the REE of CKD patients, comparing it with healthy individuals, showed that the values were reduced, similar, or increased. Furthermore, comorbidities such as diabetes, inflammation, and hyperparathyroidism contribute to increased REE in CKD.^3^ The study by Oliveira et al. provided a scarcely studied information - the comparison of REE in the pre-dialysis phase, at the beginning of dialysis and after a month of dialysis therapy in the same individuals.

Considering the limitations of single parameters, subjective global assessment and adapted models have gained notoriety for being simple and reliable tools for assessing nutritional status.^4^ In addition to these global methods, functional capacity, particularly handgrip strength,^5^ have also gained greater prominence in recent years. The relationship between global assessment models, as well as handgrip strength, with clinical outcomes has already been proven in the Brazilian population with CKD.^6^^-^ 8

The changes caused by CKD progression, especially in hormonal, inflammatory, and metabolic cascades, end up triggering a reduction in the body’s reserves of energy and protein, culminating in the development of an energy-protein malnutrition condition. The search for methodologies for tracking nutritional status in this vulnerable population is a continued effort to better nutritional and clinical management and an adequate assessment of the individual’s prognosis. Finally, longitudinal assessment is crucial in the early identification of nutritional changes in order to prevent their progression and contribute to the reduction of the high morbidity and mortality observed in CKD population.
